# RhoGEF17—An Essential Regulator of Endothelial Cell Death and Growth

**DOI:** 10.3390/cells10040741

**Published:** 2021-03-27

**Authors:** Pamina Weber, Doris Baltus, Aline Jatho, Oliver Drews, Laura C. Zelarayan, Thomas Wieland, Susanne Lutz

**Affiliations:** 1 Experimental Pharmacology Mannheim (EPM), European Center for Angioscience (ECAS), Medical Faculty Mannheim, Heidelberg University, Ludolf-Krehl-Str. 13-17, 68167 Mannheim, Germany; pamina.weber@list.lu (P.W.); doris.baltus@medma.uni-heidelberg.de (D.B.); 2 Institute of Pharmacology and Toxicology, University Medical Center Göttingen, Robert-Koch-Strasse 40, 37075 Göttingen, Germany; aline.jatho@med.uni-goettingen.de (A.J.); laura.zelarayan@med.uni-goettingen.de (L.C.Z.); 3 DZHK (German Center for Cardiovascular Research) Partner Site Göttingen, Göttingen, Germany; 4 Institute for Clinical Chemistry, Medical Faculty Mannheim, Heidelberg University, Theodor-Kutzer-Ufer 1-3, 68167 Mannheim, Germany; Oliver.Drews@umm.de; 5 DZHK (German Center for Cardiovascular Research) Partner Site Heidelberg/Mannheim, Mannheim, Germany

**Keywords:** rho guanine nucleotide exchange factor, adherens junction complex, endothelial cell, non-canonical β-catenin signaling, anchorage-dependent cell death

## Abstract

The Rho guanine nucleotide exchange factor RhoGEF17 was described to reside in adherens junctions (AJ) in endothelial cells (EC) and to play a critical role in the regulation of cell adhesion and barrier function. The purpose of this study was to analyze signal cascades and processes occurring subsequent to AJ disruption induced by RhoGEF17 knockdown. Primary human and immortalized rat EC were used to demonstrate that an adenoviral-mediated knockdown of RhoGEF17 resulted in cell rounding and an impairment in spheroid formation due to an enhanced proteasomal degradation of AJ components. In contrast, β-catenin degradation was impaired, which resulted in an induction of the β-catenin-target genes cyclin D1 and survivin. RhoGEF17 depletion additionally inhibited cell adhesion and sheet migration. The RhoGEF17 knockdown prevented the cells with impeded cell–cell and cell–matrix contacts from apoptosis, which was in line with a reduction in pro-caspase 3 expression and an increase in Akt phosphorylation. Nevertheless, the cells were not able to proliferate as a cell cycle block occurred. In summary, we demonstrate that a loss of RhoGEF17 disturbs cell–cell and cell–substrate interaction in EC. Moreover, it prevents the EC from cell death and blocks cell proliferation. Non-canonical β-catenin signaling and Akt activation could be identified as a potential mechanism.

## 1. Introduction

Endothelial cells (EC) form a continuous monolayer that covers the luminal side of blood vessels, thereby building a chemicophysical barrier between the blood stream and the interstitium. The integrity of this endothelial barrier is complexly regulated and dependent on cell–cell contacts including adherens junctions (AJ). Besides their function in cell–cell adhesion, AJ play a role in actin cytoskeleton remodeling, intracellular signaling, and transcriptional regulation in EC [1]. The major structural component of AJ in EC is vascular endothelial (VE)-cadherin. In addition, EC express neuronal (N)-cadherin, which was suggested to play a role in stabilizing contacts between EC and mural cells [2,3,4,5,6]. Of note, in addition to primary human umbilical vein EC (HUVEC) immortalized rad fad pad endothelial cells (RFPEC) were used in this study, which do not express significant levels of VE-cadherin, but use instead N-cadherin for cell–cell contacts [7]. Cadherins display a homophilic interaction on the extracellular side and are bound to and regulated by diverse proteins at their shorter intracellular C-termini. Amongst these are proteins of the *Armadillo*-repeat gene family: p120-catenin and β-catenin [8,9]. Both catenins are not only part of the AJ, but regulate in addition gene transcription. β-catenin is the critical regulator of gene expression in the canonical Wnt signaling cascade and in the absence of a stimulus its stability is regulated via the β-catenin destruction complex involving phosphorylation and ubiquitination [10,11]. When Wnt-signaling is activated, β-catenin translocates into the nucleus and regulates gene expression together with members of the TCF/LEF family of transcription factors [12,13]. This signaling is partly counteracted by p120-catenin, which interacts with the transcriptional inhibitor Kaiso, and was shown to regulate β-catenin expression and β-catenin-dependent gene expression [14,15].

Cadherins are linked to the actin cytoskeleton, involving the β-catenin binding partner α-catenin. Whether α-catenin is a direct linker or other proteins like EPLIN and vinculin are necessary, is, however, a matter of debate [16,17]. Moreover, diverse proteins involved in the regulation of the actin cytoskeleton reside in AJ or are associated to them. This includes monomeric RhoGTPases and their accessory regulators like p190RhoGAP as well as the RhoA and Rac1 activator Trio (for detailed review, see [18]). A RhoA-C activator, which has been described to be present in AJ, is the guanine nucleotide exchange factor RhoGEF17, alias tumor endothelial marker 4 (TEM4) [19]. The RhoGEF17 gene (ARHGEF17) was initially found to be up-regulated during angiogenesis in human tumor endothelium [20]. Furthermore, a point mutation in ARHGEF17, leading to premature codon termination, was found in melanoma cells [21]. RhoGEF17 belongs to the Dbl-homology (DH) protein family, comprises 2063 amino acids, and contains, beside the DH domain, an atypical pleckstrin homology (PH) domain. Within the N-terminus, a functional actin binding domain (ABD) was identified, which allows RhoGEF17 to directly bind to F-actin [22]. RhoGEF17 is expressed in a variety of tissues like in the heart, lung, and kidney and was detected in endothelial and epithelial cells, as well as in different cancer cells [19,23]. In EC, RhoGEF17 was demonstrated to localize at AJ and was shown to regulate cell migration. As a potential underlying mechanism, RhoGEF17′s ability to activate RhoA and C was discussed [19,24]. In a recent study, Yang and colleagues demonstrated that low frequency mutations in RhoGEF17 are associated with a higher risk for intracranial aneurysms in humans. Moreover, they could confirm their association by showing that a deficiency in RhoGEF17 in zebrafish results in intracranial hemorrhage [25]. Our study further extends the knowledge on RhoGEF17 in EC. We are able to demonstrate that its downregulation impairs cell–cell and cell–matrix interaction and thus spheroid formation, adhesion and migration. More importantly, we show that the cells with impaired cell-cell and cell matrix contacts are protected from apoptosis and unable to proliferate and are thus trapped between cell death and growth. On a molecular level, we found an enhanced proteasomal degradation of AJ proteins, a non-canonical β-catenin signaling, and a shift in the balance of pro- and anti-survival factors.

## 2. Materials and Methods

**Antibodies and reagents**—The following antibodies were used: rabbit anti-RhoGEF17 (ProSci, Poway, CA, USA), goat anti-pan-cadherin (Santa Cruz, Heidelberg, Germany), mouse anti-N-cadherin (BD Biosciences, Heidelberg, Germany), rabbit anti-p120-catenin (Epitomics by Abcam, Cambridge, UK), mouse anti-p120-catenin (Santa Cruz, Heidelberg, Germany), rabbit anti-β-catenin (Santa Cruz, Heidelberg, Germany), rabbit anti-β-catenin (Zymo Research, Freiburg, Germany), rabbit anti-phospho-β-catenin (Ser33/Ser37/Thr41) (Cell Signaling Technology, Frankfurt, Germany), mouse anti-histone H1 (Santa Cruz, Heidelberg, Germany), mouse anti-vinculin (Sigma-Aldrich, Taufkirchen, Germany), mouse anti-α-tubulin (Sigma-Aldrich, Taufkirchen, Germany), rabbit anti-phospho-Akt (Ser473) (Cell Signaling Technology, Frankfurt, Germany), rabbit anti-Akt (Cell Signaling Technology, Frankfurt, Germany), rabbit anti-cyclin D1 (Cell Signaling Technology, Frankfurt, Germany), rabbit anti-axin 1 (Cell Signaling Technology, Frankfurt, Germany), mouse anti-GAPDH (Meridian/Biodesign, Memphis, TN, USA), rabbit anti-survivin (Abcam, Cambridge, UK), mouse anti-VE-cadherin (Enzo Life Sciences, Lörrach, Germany), rabbit anti-caspase 3 (Biorad, Feldkirchen, Germany), rabbit anti-cleaved caspase 3 (Cell Signaling Technology, Frankfurt, Germany), mouse anti-β-actin (Sigma-Aldrich, Taufkirchen, Germany). Phalloidin (Sigma-Aldrich, Taufkirchen, Germany) was used to stain F-actin and cell nuclei were stained with DAPI (Sigma-Aldrich, Taufkirchen, Germany).

**Generation of recombinant adenoviruses**, **cell culture and transduction**—Recombinant adenoviruses were generated according to the method of He et al. [26]. The EGFP-control adenovirus was generated by recombination of the both vectors pAd-Easy-1 and pAdTrack-CMV which were kindly provided by Bert Vogelstein, Baltimore, USA. The RhoGEF17-specific short hairpin RNAs exhibited the following sequences: 5‘-CAGTGTCATTGATACAGCCAGCAAA-3‘ (sh17-1) and 5‘-GTATCTGAATAACCAGGTGTT-3‘ (sh17-2) [24]. They were cloned in a modified pAdTrack-vector, in which the expression of the shRNA was regulated by an H1-promotor. The adenovirus for RhoGEF17 additionally encodes for EGFP. The shp63RhoGEF (shp63) adenovirus was described previously [27,28].

Spontaneously immortalized microvascular EC isolated from the capillaries of rat epididymal fat tissue (RFPEC) [29] (kind gift from A. Horowitz, Dartmouth Medical School, New Hampshire, USA) were maintained in high glucose Dulbecco’s modified Eagle’s medium (DMEM; Sigma-Aldrich, Taufkirchen, Germany) supplemented with 10% (*v*/*v*) heat-inactivated bovine calf serum (FCS; Cytiva, Marlborough, MA, USA), 100 U/mL penicillin (Sigma-Aldrich, Taufkirchen, Germany), 100 µg/mL streptomycin (Sigma-Aldrich, Taufkirchen, Germany) and 2 mM L-glutamine (Sigma-Aldrich, Taufkirchen, Germany) at 37 °C in an atmosphere of 5% CO_2_.

Macrovascular EC isolated from human umbilical cord (HUVEC) were prepared as described previously [30] and cultured in endothelial growth medium (C-22110, Promocell, Heidelberg, Germany) containing supplements (C-39210, Promocell, Heidelberg, Germany) and 2% (*v*/*v*) fetal bovine serum (FCS) (C-37350, Promocell, Heidelberg, Germany) in a humidified atmosphere containing 5% CO_2_ at 37 °C.

RFPEC and HUVEC were seeded in cell culture dishes, coated with collagen I (BD Bioscience) or gelatin (Sigma-Aldrich, Taufkirchen, Germany), respectively, and cultured in the corresponding growth medium. At a confluence of 70% to 80%, cells were transduced with the adenoviral constructs (multiplicity of infection (MOI) of 5000 to 50,000) to obtain a transduction rate of 70% to 100% after 48 h. All experiments were performed after 24 to 48 h post transduction.

The treatment with 100 nM Bortezomib (Selleck Chemicals, Houston, TX, USA), which was dissolved in DMSO, was always carried out with equal volumes of DMSO in the control transduced or non-transduced cells.

**Spheroid formation**—Spheroids were generated from 400 RFPECs and embedded into a 3D collagen-based gel placed in a 48-well plate as described before [31].

**Cell fractionation**—For cell fractionation, RFPEC seeded on collagen I-coated 10 cm dishes were scraped off in ice-cold TE buffer and homogenized on ice. After centrifugation at 13,000× *g* the cell pellet containing nuclear fraction was resuspended in the TE buffer, homogenized, and mixed with 1% Triton X-100. The supernatant was homogenized and ultracentrifuged at 100,000× *g*. The supernatant containing the cytosolic cell fraction was supplemented with 1% Triton X-100. All fractions were prepared for SDS-PAGE.

**qRT-PCR**, **immunoblotting**, **and immunofluorescence**—For quantitative reverse transcription PCR (qRT-PCR), total RNA was isolated using RNeasy Mini Kit (Qiagen, Hilden, Germany) according to the manufacturer’s instructions. The RevertAid™ first strand cDNA synthesis kit (Fermentas, Sankt Leon-Rot, Germany) was used for reverse transcription and cDNA was amplified using the EvaGreen dye for qRT-PCR (Solis Biodyne, Tartu, Estonia) according to the manufacturer’s protocol for 40 cycles. The following oligonucleotides were synthesized by Eurofins/MWG (Ebersberg, Germany): N-cadherin: forward: 5′-tggcggccttgcttcaggcatctct-3′, reverse: 5′-gcgtacactgtgccgtcctcatcca-3′; PBGD: forward: 5′-cctgaaactctgcttcgctg -3′, reverse: 5′-ctggaccatcttcttgctgaa-3′. For the amplification of RhoGEF17 and p120-catenin, the following QuantiTect primers (Qiagen, Hilden, Germany) were used: RhoGEF17: QT00446439 and p120-catenin: QT01612415. The quantification was performed with the standard curve method and the following cDNA dilutions of a reference cDNA: 1:10, 1:30, 1:100, 1:300. All measurements were performed in quadruplicates in a 384-well plate.

For immunoblotting, cell lysates were prepared using RIPA buffer, resolved using SDS-PAGE (6% to 12% acrylamide), transferred onto a nitrocellulose membrane, probed with primary and appropriate secondary antibodies (Sigma-Aldrich, Taufkirchen, Germany), detected with enhanced chemoluminescence (Lumi Light, Roche, Mannheim, Germany; Super Signal West Femto, Pierce) and documented with an imaging system.

For immunofluorescence, cells were fixed with a fixation solution of 4% PFA and methanol (Roth) and permeabilized with 0.05% Triton X-100 in PBS. Proteins were probed with the respective primary antibodies, followed by incubation with Alexa Fluor- (Dianova, Hamburg, Germany), DyLight- (Rockland, Limerick, PA, USA) or Cy3-conjugated (Dianova, Hamburg, Germany) secondary antibodies. Images were acquired with an Olympus inverted fluorescence microscope.

**Analysis of cell adhesion**—For the analysis of cell adhesion, adenoviral-transduced RFPEC were trypsinized and resuspended in growth medium. Half of the cell suspension was transferred into a new 6-well plate coated with collagen I and incubated at 37 °C with 5% CO_2_. After 1, 3, 6, 12, and 24 h, cell adhesion as well as cell surface were documented by bright field and fluorescence microscopy (Olympus, Hamburg, Germany). Per condition and time point ten pictures were randomly taken and the percentage of transduced adherent and non-adherent cells was determined by detection of EGFP. The surface of the cells was measured with the software Cell^M^ from Olympus (Hamburg, Germany).

**Analysis of cell migration**—For the scratch assay, adenoviral-transduced RFPEC were cultured until confluence was reached. The monolayer was scratched with a P1000 pipette tip to induce a “wound”. Sheet migration was measured by imaging with an inverted fluorescence microscope (Olympus, Hamburg, Germany) after 0, 3, 6, 12, and 24 h. Ten pictures of the scratch were randomly taken per condition and time point and the distance between the border of the visual field and the cell sheet was determined, and cells were counted using the software Cell^M^ from Olympus (Hamburg, Germany). In addition the number of single cells in the “wound” was counted.

**Annexin V staining**—RFPEC were seeded and transduced in 24-well culture plates coated with collagen I. 48 h post transduction; the cells were washed with pre-warmed PBS and incubated with Alexa Fluor 594-conjugated annexin V (Life Technology, Frankfurt, Germany) for 15 min. The percentages of EGFP^+^/Annexin V^+^ (adenovirally transduced and apoptotic) and EGFP^-^/Annexin V^+^ (not transduced and apoptotic) cells were evaluated by fluorescence microscopy (Olympus, Hamburg, Germany).

**Cell cycle analysis**—For cell cycle analysis, cells were adenoviral-transduced for 48 h and fixed with ethanol. RNA was removed by treatment with ribonuclease (Qiagen, Hilden, Germany). DNA staining was performed with propidium iodide (Invitrogen) and cells within distinct cell cycle phases were analyzed by flow cytometry measurement. All samples were analyzed on FACSCalibur flow cytometry (BD Biosciences, Heidelberg, Germany), and the data were processed using FACSDiva™ software (BD Biosciences, Heidelberg, Germany).

**Analysis of cell proliferation**—RFPEC were seeded and transduced in collagen I-coated 96-well culture plates. The cells were fixed and permeabilized 24, 36, and 48 h post transduction. After incubation with 1 μg/mL DAPI in Roti-Block (Carl Roth, Karlsruhe, Germany) for 1 h at RT in the dark, the cell number was evaluated using the Cellavista system (Synentech, Elmshorn, Germany). HUVEC were seeded in gelatin-coated 24-well plates and transduced 24 h later. After a further 24, 48, and 72 h bright field and EGFP fluorescence images were taken with a microscope and the cells (EGFP^-^ and EGFP^+^) were manually counted.

**Statistics**—The statistical analysis was performed using Graph Pad Prism 5 or 7. All results are given as mean+SEM. To test for normal distribution, the F-test was used. Data of two groups were compared using the Student’s t-test. The analysis and comparison of three and more groups was performed with the one or two way analysis of variance (ANOVA) and the corresponding post-test. *p*-values ≤ 0.05 were considered statistically significant and are indicated.

## 3. Results

### 3.1. RhoGEF17 Is Essential for AJ Formation in EC

**Figure 1:**
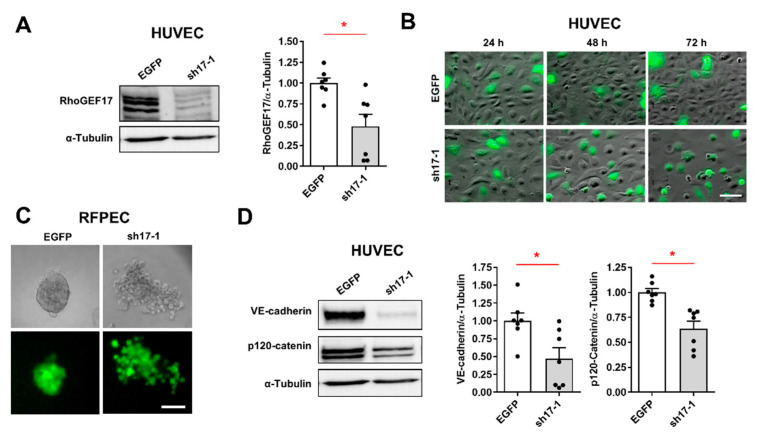
RhoGEF17 is essential for cell-cell contacts and adherens junctions (AJ) protein regulation in EC. (**A**,**B**,**D**) Human umbilical vein endothelial cells (HUVEC) were transduced with adenoviruses encoding EGFP alone (EGFP) or in addition to a shRNA against RhoGEF17 (sh17-1). (**A**) After 48 h, RhoGEF17 was detected in cell lysates. Shown are representative immunoblots of RhoGEF17 and α-tubulin and the quantitative analysis. Values are normalized and given as means + SEM with the single data points, n = 7, * *p* < 0.05 analyzed by paired t-testing. (**B**). Depicted are bright field/EGFP overlay images of transduced HUVEC. Scale bar = 100 µm. (**C**) Rat fat pad endothelial cells (RFPEC) were transduced for 48 h and then used to generate spheroids. Bright field and fluorescence images are shown. Scale bar = 200 µm. (**D**) VE-cadherin, p120-catenin, and α-tubulin were detected by immunoblot in lysates of transduced HUVEC. Shown are representative immunoblots and the quantified data normalized by α-tubulin and relative to EGFP as means + SEM with the single data points, n = 4 − 7, * *p* < 0.05 analyzed by paired t-testing.

In order to investigate the involvement of RhoGEF17 in AJ signaling, we first verified its role in the regulation of AJ integrity. We transduced HUVEC and spontaneously immortalized RFPEC [29], with adenoviruses encoding two shRNAs (sh17-1, sh17-2) targeting RhoGEF17 in its PH domain (Supplementary Materials Figure S1A). For negative control, we used an EGFP-expressing adenovirus (EGFP) and an adenovirus encoding a shRNA against p63RhoGEF (shp63) [27,28], which is not expressed in EC [32]. We show that a downregulation of RhoGEF17 by around 50% in HUVEC and 70% in RFPEC resulted in a gradual cell rounding and loss of cell–cell contacts over time (Figure 1A,B, Supplementary Materials Figure S1B–D). We substantiated this finding by demonstrating that after the knockdown of RhoGEF17 no RFPEC spheroids were formed (Figure 1C, Supplementary Materials Figure S1E). In contrast to what has been shown before, the AJ proteins VE-cadherin and N-cadherin, the dominant isoforms in HUVEC and RFPEC [7], respectively, and p120-catenin were down-regulated in sh17-transduced cells compared to both controls (Figure 1D, Supplementary Materials Figure S1F,G). Taken together this data indicates that a loss of RhoGEF17 in EC results in a loss of AJ proteins and consequently in an impairment of cell–cell interaction.

### 3.2. AJ Proteins Are Predominantly Lost by Proteasomal Degradation in EC with Low Expression of RhoGEF17

**Figure 2:**
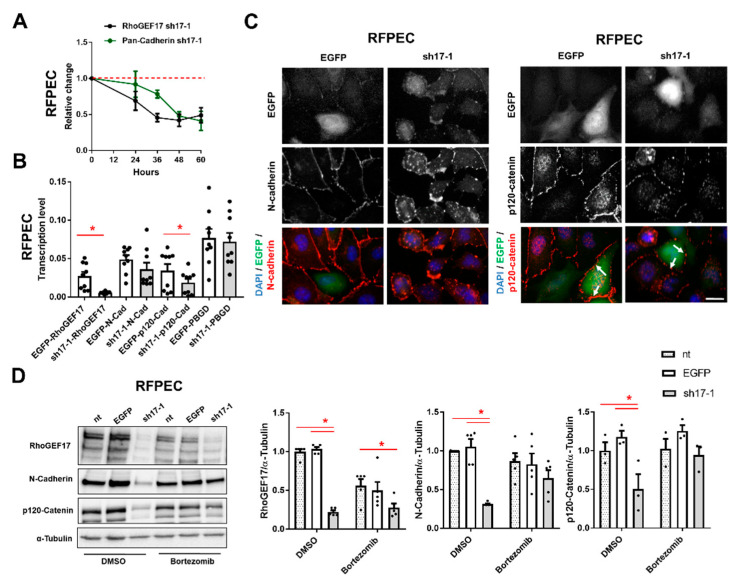
Loss of RhoGEF17 results in the proteasomal degradation of AJ proteins in RFPEC. RFPEC were transduced with adenoviruses encoding EGFP (EGFP), or EGFP and a shRNA against RhoGEF17 (sh17-1). (**A**) Immunoblot analysis of RhoGEF17, pan-cadherin and α-tubulin was performed at the indicated time points. The values were normalized by α-tubulin and are given as means ± SEM relative to the time point 0 h, n = 3–21. (**B**) Immunofluorescence analysis was performed 48 h after transduction. Depicted are EGFP images, immunofluorescence staining of N-cadherin (left), p120-catenin (right) and the overlays with DAPI. Scale bar = 20 µm. (**C**) The transcript levels of RhoGEF17, N-cadherin, p120-catenin and the housekeeping gene PBGD were determined by qPCR after 48 h of transduction. The values are given as means + SEM with the single data points, n = 9, * *p* < 0.05 assessed by paired t-testing. (**D**) The proteasome was inhibited with 100 nM Bortezomib for 2 h. Non-transduced (nt) cells were used as additional control. RhoGEF17, N-cadherin and p120-catenin were detected by immunoblot in whole cell lysates. Shown are representative immunoblots of RhoGEF17, N-cadherin, p120-catenin and α-tubulin (left) and the quantitative analyses. Values were normalized by α-tubulin and are given relative to non-transduced cells treated with DMSO only. Shown are means + SEM and the single data points; n = 3–5, * *p* < 0.05 assessed by 2-way ANOVA with Tukey’s multiple comparison testing.

Following, we investigated the mechanism behind the down-regulation of AJ proteins as a consequence of the RhoGEF17 knockdown. We used for this purpose RFPEC, which showed comparable results to HUVEC with respect to cell rounding and protein regulation, but could be more efficiently transduced. We demonstrated by a time course that the decline in cadherin protein levels was delayed by around 12 h compared to RhoGEF17 expression (Figure 2A). To test if this results from a transcriptional change, we performed a qPCR analysis. The knockdown of RhoGEF17 resulted in an expected strong decrease in RhoGEF17 mRNA and a moderate decrease in p120-catenin mRNA. However, neither N-cadherin nor the housekeeping gene PBGD showed any differences in transcript levels (Figure 2B). Next, we detected N-cadherin and p120-cateinin by immunofluorescence, which demonstrated that both proteins were not only localized at the plasma membrane, but could be additionally found in a punctated pattern inside the cells when RhoGEF17 expression is reduced (Figure 2C). As these findings suggested that the decline in N-cadherin protein was not based on a transcriptional change, but on a change in protein turnover, we treated the transduced cells with the proteasome inhibitor Bortezomib and performed an immunoblot analysis. Indeed, after inhibition of the proteasome, no differences in the protein content of N-cadherin could be detected in RhoGEF17-knockdown cells compared to control. Likewise, the drop in p120-catenin was prevented by Bortezomib, suggesting that proteasomal degradation and not the decline in gene transcription is the dominant mechanism leading to the p120-catenin downregulation (Figure 2D).

### 3.3. The Reduction of RhoGEF17 Is Accompanied by an Increased Protein Modification of β-Catenin and Changes in the Expression of Associated Genes

**Figure 3:**
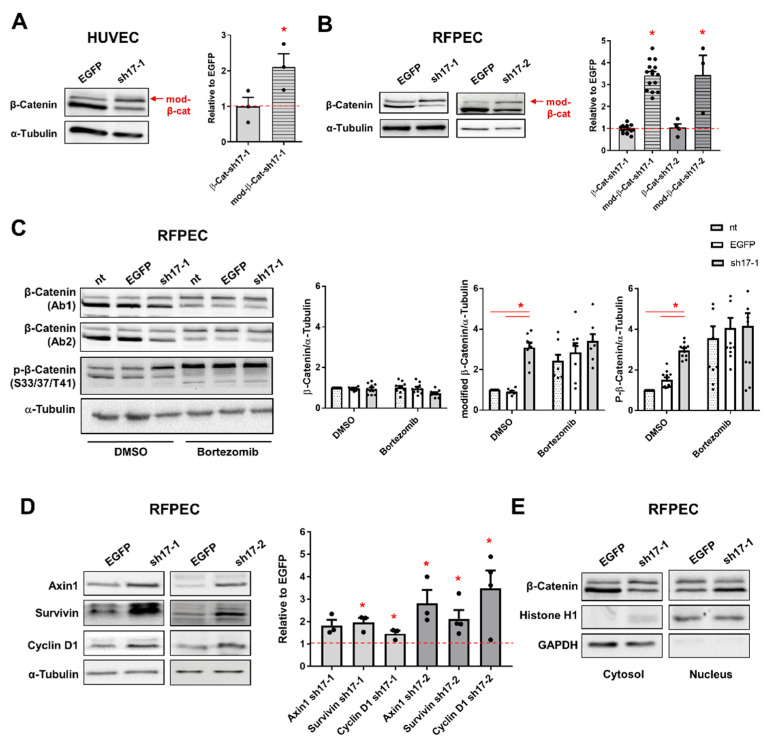
Loss of RhoGEF17 results in the accumulation of phosphorylated β-catenin and increases β-catenin target gene expression in EC. (**A**) HUVEC or (**B**) RFPEC were transduced for 48 h. Representative immunoblots of β-catenin and α-tubulin are shown. The intensity of both detected β-catenin bands (β-cat) as well as the intensity of the upper β-catenin band (mod-β-cat) were quantified and normalized by α-tubulin. The values are given relative to the EGFP control as means + SEM with the single data points, n = 3 (HUVEC), n = 3–14 (RFPEC), * *p* < 0.05 vs. EGFP control (not shown) assessed by paired t-testing. (**C**) The proteasome was inhibited with 100 nM Bortezomib for 2 h in RFPEC. β-catenin and phosphorylated β-catenin were detected by immunoblot. Shown are representative immunoblots of β-catenin (Ab1 = Santa Cruz, Ab2 = Zymo Research), *p*-β-catenin (S33/37/T41) and α-tubulin (left) and the quantitative analyses. The values are normalized and given as means + SEM with the single data points, n = 8–11, * *p* < 0.05 assessed by 2-way ANOVA with Tukey’s multiple comparison test. (**D**) Axin1, survivin, cyclin D1 and α-tubulin were detected by immunoblot in RFPEC lysates. Shown are representative immunoblots (left) and the corresponding analyses. The values are given relative to the EGFP control as means + SEM with the single data points, n = 3, * *p* < 0.05 vs. EGFP control (not shown) assessed by paired t-testing. (**E**) Cell fractionation experiments were performed with transduced RFPEC. β-catenin, histone H1 and GAPDH were detected by immunoblot in different cell fractions.

AJ dissociation results in non-canonical Wnt signaling by the release of the major Wnt pathway mediator β-catenin [33]. Therefore, we studied the impact of the RhoGEF17 knockdown on β-catenin by immunoblot analysis. We detected two distinct β-catenin signals of which the signal with the higher molecular weight, named as modified β-catenin (mod. β-catenin), was increased after depletion of RhoGEF17 in HUVEC and RFPEC (Figure 3A,B, Supplementary Materials Figure S2A). The total β-catenin content (intensity of both signals together) was, however, unchanged (Figure 3A,B). In order to characterize the nature of the two β-catenin variants, we treated RFPEC with Bortezomib. Inhibition of the proteasome had no impact on total β-catenin levels, but shifted the ratio of both variants in favor of the larger one. We further used a phospho-specific anti-β-catenin antibody recognizing its phosphorylation at serine 33, serine 37 and threonine 41. This allowed us to demonstrate that inhibition of the proteasome, and more importantly RhoGEF17 depletion, resulted in an accumulation of phosphorylated β-catenin (Figure 3C). We next analyzed the impact of the RhoGEF17 knockdown on the expression of the inhibitory Wnt pathway component axin1 and the two downstream targets survivin and cyclin D1. Surprisingly, we found all three proteins to be increased after the knockdown of RhoGEF17 (Figure 3D). We further verified for axin1 and cyclin D1 that this increase was due to an enhanced transcription (Supplementary Materials Figure S2B). As this implied that despite the increase in β-catenin phosphorylation and the higher expression of the Wnt inhibitor axin1, a net activation of the Wnt pathway had occurred, we investigated the distribution of β-catenin in the cytosolic and nuclear fractions of the transduced RFPEC. In line, we found more unmodified, transcriptional active β-catenin in the nuclear fraction of RhoGEF17 knockdown cells compared to control transduced cells, whereas in the cytosolic fraction the opposite distribution was detectable (Figure 3E).

### 3.4. The Reduction of RhoGEF17 Impairs RFPEC Adhesion and Sheet Migration

**Figure 4:**
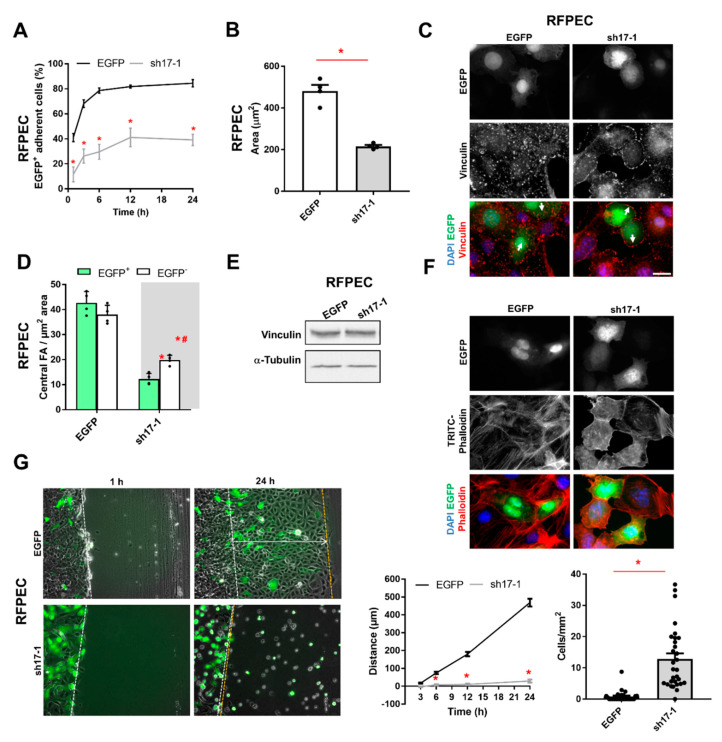
The RhoGEF17 knockdown alters the adhesion and migration behavior of RFPEC. RFPEC were transduced for 48 h. (**A**) The cells were detached and reseeded. Adhesion was monitored by fluorescence microscopy over a time course of 24 h. Depicted are the percentages of transduced (EGFP^+^), adherent cells given as means ± SEM, n = 6, * *p* < 0.05. (**B**) The surface area of the cells was determined at the end (24 h) of the adhesion assay. Given are the means + SEM with the single data points, n = 4, * *p* < 0.05 assessed by t-testing. (**C**) Shown are EGFP images, immunofluorescence staining of vinculin and the merges of EGFP (green), vinculin (red), and DAPI (blue). Scale bar = 20 µm. (**D**) The quantification of the number of central focal adhesions in transduced (EGFP^+^) and non-transduced (EGFP^-^) adjacent cells are given as means + SEM with the single data points, n = 4, *p* < 0.05, * vs. EGFP-transduced, ^#^ EGFP^+^ vs. EGFP^-^ in sh17-1 transduced cells. (**E**) Vinculin was detected by immunoblot. Shown are representative immunoblots of vinculin and α-tubulin. (**F**) Fluorescence imaging of the transduced cells was performed. Shown are EGFP and TRITC-phalloidin images together with the merges of EGFP (green), phalloidin (red), and DAPI (blue). Scale bar = 20 µm. (**G**) Confluent transduced cells were scratched and imaged at the indicated time points. Left: Representative bright field/fluorescent images are show. Middle: The migration distance of the sheet was measured as indicated in the left images by the arrow. Given are the quantified data as mean ± SEM, n = 3 with 10 replicates per experiment, * *p* < 0.05 vs. EGFP assessed by 2-way ANOVA with Sidak’s multiple comparison test. Single, EGFP^+^ cells in the wound were counted at the end of the assay. The number of cells per mm^2^ are given as means + SEM of all measured 30 wells, * *p* < 0.05 assessed an unpaired t-test.

Based on the observed cell rounding of RhoGEF17 knockdown cells, we analyzed next the adhesion behavior of transduced RFEPC and could show that the knockdown of RhoGEF17 strongly impaired the adhesion ability (Figure 4A, Supplementary Materials Figure S3A) and maximal cell spreading reflected by the reduced cell area (Figure 4B, Supplementary Materials Figure S3B). By staining of the focal adhesion protein vinculin, we were able to demonstrate that the knockdown of RhoGEF17 resulted in a loss of central focal adhesion sites in transduced (EGFP^+^) cells, but also in non-transduced (EGFP^-^) adjacent cells (Figure 4C,D). The expression level of vinculin was unaltered (Figure 4E). We could further show that the loss in focal adhesion sites was accompanied by a loss in prominent actin fibers (Figure 4F). As cell adhesion and migration are strongly connected processes, we studied next the influence of the RhoGEF17 knockdown on sheet migration. The work presented by Mitin and colleagues in 2013 already demonstrated that a knockdown of RhoGEF17 impairs the migration directionality of EC in a single cell migration assay [24]. We found that the knockdown of RhoGEF17 fully impaired the coordinated closure of the cell-free area in a scratch assay. Interestingly, more single cells were present in the “wound” (Figure 4G, Supplementary Materials Figure S3C).

### 3.5. The Reduction of RhoGEF17 Prevents Apoptosis and Induces a Cell Cycle Block in EC

**Figure 5:**
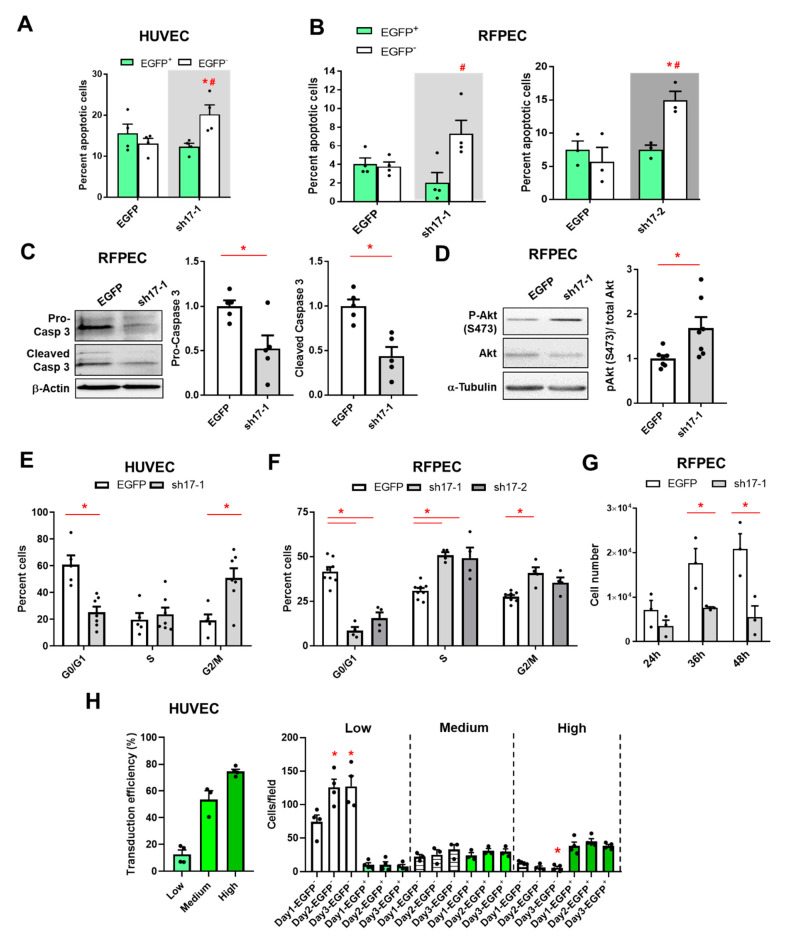
The reduction of RhoGEF17 prevents apoptosis and induces a cell cycle block in EC. HUVEC (**A**,**E**,**H**) or RFPEC (**B**–**D**,**F**,**G**) were transduced. (**A**,**B**) Apoptotic cells were detected by annexin-V in semi-efficiently transduced cells after 48 h. The number of apoptotic transduced (EGFP^+^) and non-transduced (EGFP^-^) adjacent cells are given as means + SEM with the single data points, n = 3–4, *p* < 0.05 * vs. EGFP-transduced, ^#^ EGFP^+^ vs. EGFP^-^ in sh17 transduced cells assessed by 1-way ANOVA with Tukey’s multiple comparison test. **C**) Pro- and cleaved caspase 3 expression was analyzed by immunoblot in 100% transduced cells after 48 h. Representative immunoblots of both variants and β-actin (**B**) and the analyses (**C**) are shown. Quantified values are normalized by β-actin and are given relative to EGFP. Shown are the means + SEM with the single data points, n = 5, * *p* < 0.05 assessed by paired t-test. (**D**) Phosphorylation of Akt was detected in whole cell lysates by immunoblot 48h after transuction. Shown are representative immunoblots of pAkt (S473), total Akt and α-tubulin and the analysis. Quantified p-Akt was normalized by Akt and is given relative to EGFP. Shown are the means + SEM with the single data points, n = 7, * *p* < 0.05 assessed by paired t-test. (**E**,**F**) Cell cycle progression was analyzed by flow cytometry in cells transduced for 48 h. Calculation of G1, S, and G2/M phases are given as means + SEM with the single data points, n = 4–8, * *p* < 0.05 assessed by 2-way ANOVA with Sidak’s or Tukey’s multiple comparison test. (**G**) RFPEC were 100% transduced and replated. After the indicated time points cell proliferation was assessed by automated nuclei counting. The absolute cell number is given as means + SEM with single data points, n = 3, * *p* < 0.05 assessed by 2-way ANOVA with Sidak’s multiple comparison test. (**H**) HUVEC were seeded and transduced with different amounts of viruses in 24-well plates. One, two, and three days later images were taken and the non-transduced (EGFP^-^) and transduced (EGFP^+^) cells were counted manually in 160x magnification images. Left: The results were clustered according to the transduction efficiencies in low, medium, and high transduction experiments. Right: Given are the number of cells per field of view in the different conditions, n = 3–4, * *p* < 0.05 vs. the corresponding Day1 data as detected by 1-way ANOVA with a Dunnett’s multiple comparison test.

Adherent cells, like EC, which experience disturbances in their cell-cell and cell-matrix contacts undergo anchorage-dependent apoptosis, also termed anoikis [34]. We wondered if the knockdown of RhoGEF17 and thus the disruption of AJ and the weakening of focal adhesions consequently result in a higher apoptosis rate. We first performed an annexin V staining of semi-efficiently transduced cells and counted EGFP^+^/annexin V^+^ and EGFP^-^/annexin V^+^ cells. We show that the percentages of apoptotic transduced HUVEC and RFPEC did not differ between the conditions, but for the non-transduced cells significant higher percentages of apoptotic cells were found in the conditions were the sh17 viruses were used. In general, the basal apoptosis rate of HUVEC was higher as of the immortalized RFPEC (Figure 5A,B, Supplementary Materials Figure S4A). This data indicated that a knockdown of RhoGEF17 does not only impair cell-cell and cell-matrix contacts, but surprisingly also protect the rounded knockdown cells from apoptosis. To further validate this unexpected finding, we used 100% transduced RFPEC and measured the expression of pro-caspase 3 and cleaved caspase 3 in lysates by immunoblot. In line with the annexin V staining, we found a lower expression of pro-caspase 3 and fewer cleaved caspase 3 in RFPEC with lowered RhoGEF17 expression compared to controls (Figure 5C, Supplementary Materials Figure S4B). Moreover, the pro-survival kinase Akt displayed a higher degree of phosphorylation in these cells (Figure 5D). We wondered whether the reduction in RhoGEF17 influences the cell cycle activity and proliferation capacity of the transduced cells. Therefore, cell cycle progression was investigated by flow cytometry experiments with propidium iodide (PI)-stained EC. The knockdown of RhoGEF17 in HUVEC resulted in a higher proportion of cells in G2/M phase (Figure 5E, Supplementary Materials Figure S4C) and in RFPEC more cells were found to be in S- and G2/M-phase (Figure 5F, Supplementary Materials Figure S4D). Finally, we were able to demonstrate that the down-regulation of RhoGEF17 in EC inhibited cell proliferation. For RFPEC we show that an efficient RhoGEF17 knockdown inhibits proliferation compared to EGFP-transduced cells (Figure 5G). Importantly, we transduced HUVEC with varying amounts of the sh17-1 adenovirus to obtain low, medium, and high transduction efficiencies (12.6%, 53.6%, and 74.5%). We found that the number of EGFP^+^ cells stayed constant over two days independent of the transduction efficiency, thus demonstrating that no proliferation of cells with reduced RhoGEF17 expression occurred. We further counted the EGFP^-^ cells in these experiments, which demonstrated that in the presence of only a minor portion of EGFP^+^ cells (Low), the EGFP^-^ cells proliferated similar to the shp63 control. However, in the presence of higher numbers of EGFP^+^ cells, the proliferation of EGFP^-^ cells was blunted for medium transduction efficiency conditions and the number of non-transduced cells even decreased when the RhoGEF17 knockdown cells constituted the majority of cells (Figure 5H, Supplementary Materials Figure S4E). Therefore, this data supports not only the RFPEC proliferation data, but also our apoptosis data, demonstrating that RhoGEF17 knockdown cells were protected from apoptosis, whereas the by neighboring RhoGEF17 knockdown cells isolated wildtype cells underwent apoptosis.

## 4. Discussion

RhoGEF17 was described as a RhoA, B, and C-specific GEF [22], which was first identified in 2002 as a member of the Dbl family of RhoGEFs [23]. In EC, RhoGEF17 was found amongst the highest expressed RhoGEFs and implicated to play a role in cancer-associated angiogenesis [20,35]. In HUVEC, it was shown that a knockdown of RhoGEF17 impaired AJ integrity, the formation of tube-like structures in an in vitro angiogenesis assay, and the persistence of single cell migration [19,24].

With our study we confirmed the role of RhoGEF17 in the regulation of AJ and in cell migration, however, the strength of the phenotype was more pronounced compared to the previously published studies [19,24]. We have not only seen an impairment in AJ function, but we found that the knockdown of RhoGEF17 led to a loss of cell–cell interaction in 2D and 3D cultures. On a molecular level, we could prove that AJ proteins were down-regulated by proteasomal degradation. This included N-cadherin in RFPEC, which do not express VE-cadherin and were shown to use instead N-cadherin for EC-EC interaction [7], and VE-cadherin in HUVEC as well as p120-catenin in both cell types. Although, HUVEC do express both VE- and N-cadherin, we did not investigate changes in N-cadherin, as this cadherin isoform was suggested to play a role in heterocellular contacts, like between EC and mural cells [3,4,5,6].

Furthermore, in our experiments we found a strongly impaired cell adhesion capacity and the RhoGEF17 knockdown cells were completely unable to migrate in a sheet. As a possible explanation for the differences in phenotype severity, the methods of transduction could be accounted. Both Ngok and Mitin and coworkers used a lentiviral approach, whereas we used an adenoviral approach. In fact, it has been shown that adenoviruses are more efficient in gene transfer for EC than lentiviruses, suggesting that the achieved knockdown in our study was more pronounced and likely faster in onset [36,37].

Beyond RhoGEF17′s role in the regulation of AJ integrity, cell adhesion, and migration, we describe in our study its role in cell death, cell cycle control, and proliferation. The survival and growth of EC is strongly dependent on cell–cell and cell–matrix contacts. With respect to cell–cell contacts, it was shown that the deficiency of VE-cadherin, its cytosolic truncation, and the interference of its interaction by monoclonal antibodies results in endothelial cell apoptosis [38,39]. A loss in EC-matrix contacts also induced apoptosis, as demonstrated by exposing EC to non-adhesive surfaces, or by using antagonizing antibodies against integrins and RGD mimics [40,41,42,43]. In line with these observations, we found that EC, treated with, but not transduced by the sh17 adenovirus, showed a higher apoptosis rate as EC with cell–cell and cell–matrix contacts under control conditions. However, rounded EC with a reduction in RhoGEF17 expression did not demonstrate an increased apoptosis rate, suggesting that RhoGEF17 is involved in anoikis in EC. On a molecular level we demonstrated that RhoGEF17 knockdown cells expressed more survivin, less pro- and cleaved caspase 3, and showed an increase in Akt phosphorylation, which reflects together an anti-apoptotic phenotype. These changes are typical for anoikis-resistent cells, like metastatic cancer cells [44,45]. Also, for anoikis-resistent EC it has been described that an increase in Akt phosphorylation is essential for cell survival [34,46]. However, in contrast to anoikis-resistent EC, which are able to proliferate, RhoGEF17-depleted EC showed no proliferation capacity at all. Cell cycle analysis revealed that the fraction of RFPEC and HUVEC in G1 phase were strongly reduced and instead the percentages of cells in S- and/or G2/M-phase were increased. This data argues for a block in cell cycle, which consequently impairs proliferation. In accordance to the lower number of cells in the G1 phase, we could show that the cell cycle regulator cyclin D1 was increased. The induction of cyclin D1, and its binding to CDK4 or CDK6, was demonstrated to be a rate-limiting event during cell–cycle progression through G1 phase [47,48]. However, the exact mechanisms behind the arrest is not clear. As an explanation, a blockade of the proteasome, due to the sudden increase in substrate load by AJ disruption, could serve. For HUVEC it was demonstrated that proteasome inhibition with Bortezomib resulted in G2/M arrest and subsequently in cell death [49]. In addition, RhoGEF17 was described to be part of the spindle assembly checkpoint complex. Its depletion in HeLa cells resulted in an acceleration of mitosis, which impaired chromosome congression, biorientation, and segregation [50]. It could therefore be speculated that this novel role of RhoGEF17 finally induces a blockade of the cell cycle.

**Figure 6:**
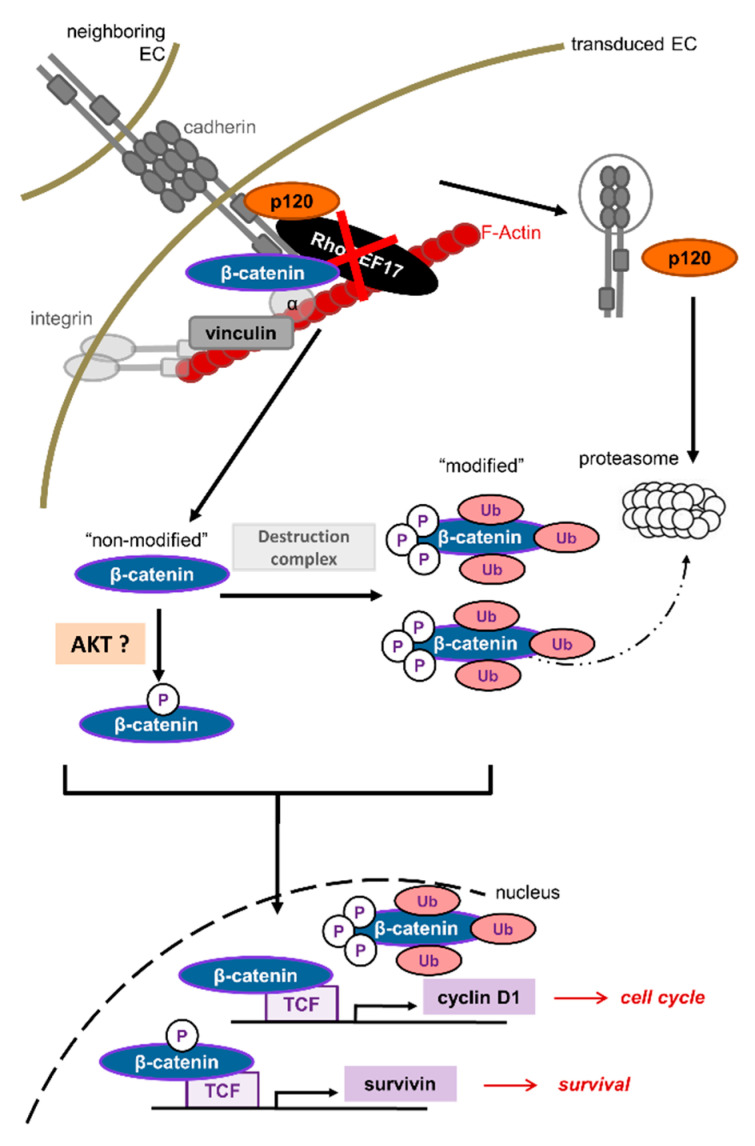
Scheme of RhoGEF17 function in EC. RhoGEF17 stabilizes AJ in EC. Its loss leads to AJ protein degradation via the proteasome and an accumulation of β-catenin in its destruction complex (phosphorylated and ubiquitinated) form. A part of the β-catenin pool, which might be phosphorylated by Akt, is translocated to the nucleus and induces β-catenin/TCF dependent gene transcription of, for example, survivin and cyclin D1. Besides impaired cell adhesion and migration due to the disruption of the AJ, RhoGEF17-depleted EC can escape anoikis and end in cell cycle arrest. In contrast neighboring cells, without RhoGEF17 knockdown, enter apoptosis due to the loss of cell–cell contacts. EC = Endothelial cell, RhoGEF17 = Rho-specific guanine nucleotide exchange factor 17, p120 = p120-catenin, α = α-catenin, TCF = Transcription factor, Ub = ubiquitinated, P = phosphorylated.

A possible signaling hub between RhoGEF17 and the described functional changes could be the multifunctional protein β-catenin. When RhoGEF17 is less expressed and cadherins are degraded, the bound junctional β-catenin is released and becomes part of the β-catenin signaling pool. This led in our study to an up-regulation of the two β-catenin target genes cyclin D1 and survivin [51,52]. Interestingly, in our immunoblot studies we detected two β-catenin bands of which we named the variant with the higher molecular weight “modified” β-catenin. Under control conditions the ratio of this modified variant compared to the variant with the lower molecular weight was around one to four and in RhoGEF17 knockdown cells the ratio was changed to one to two. When the cells were treated with Bortezomib the amount of modified β-catenin increased in the controls and was then similar under all conditions. This implies that in RhoGEF17 knockdown cells an accumulation of β-catenin takes place which can escape from proteasomal degradation. We further show that the modified β-catenin can be detected by a phospho-β-catenin antibody. Whether or not the RhoGEF17-dependent shift in β-catenin size results from phosphorylation and/or other modifications is not clear [53,54,55]. At least the shift size of around 20 kDa is indicative that larger modifications, like ubiquitination, are contributing to it [56,57]. Therefore, our data indicate that a RhoGEF17 knockdown in EC results in an accumulation of posttranslationally modified β-catenin (Figure 6). The most likely explanation for this is an overload of the proteasome by the massive degradation of other destabilized AJ proteins. In general, an increase in the cytosolic pool of β-catenin is thought to be associated with enhanced nuclear translocation and thus enhanced transcription of β-catenin dependent genes. In line with this interpretation, we detected an increase in the nuclear pool of the unmodified β-catenin as well as an upregulation of β-catenin dependent gene products like cyclin D1 and survivin after RhoGEF17 depletion. In this context, the increased activity of Akt in RhoGEF17 knockdown cells might be of importance as it phosphorylates β-catenin at serine 552 (Figure 6). This leads to β-catenin stabilization via binding to the scaffold protein 14-3-3ζ. It was further hypothesized that this phosphorylation induces a conformational change disengaging the binding of β-catenin from the destruction complex and resulting in an increased transcriptional activity, independent of GSK3β-mediated phosphorylation [58,59].

In conclusion, we show that RhoGEF17 is not only an integral part of the AJ complex in EC and thus contributes to the maintenance of cell–cell contacts and to cell migration, but has also a so far unknown role in the regulation of cell death and growth. Whether this mechanism applies only for conditions resulting from an accidental loss of RhoGEF17, as rarely found in patients with intracranial aneurysms, or exists as a phenotypic state, remains to be investigated. Based on the cellular phenotype, RhoGEF17 downregulation could play a role in, for example, EC senescence or as an intermediate step in endothelial to mesenchymal transition (EndMT). Typical features of senescent EC, which resembles the phenotype of RhoGEF17 knockdown EC, are a reduction in AJ integrity, higher expression of cyclin D1, an enhanced phosphorylation of Akt, and lowered proliferation and migration capacities. However, the cell cycle block in senescent EC occurs in G1 phase and the cells possess a larger cell surface, which contradicts our findings [60,61,62,63]. In EndMT, a loss in AJ proteins is a common feature and the signaling involves β-catenin and Akt phosphorylation. Moreover, it is likely that during EndMT the cells lose their ability to migrate in a sheet, but instead migrate as single cells as seen for RhoGEF17 knockdown cell [64,65]. Targeted approaches are necessary to gain deeper insight in RhoGEF17′s regulation and function in vivo. 

## Data Availability

Not applicable.

## Supplementary Materials

The following are available online at https://www.mdpi.com/article/10.3390/cells10040741/s1, Figure S1: RhoGEF17 is essential for cell-cell contacts and AJ protein regulation in EC; Figure S2:RhoGEF17 regulates β-catenin; Figure S3: The shp63 adenovirus has no effect on RFPEC adhesion, cell size, and sheet migration; Figure S4: The shp63 adenovirus has no effect on EC apoptosis, caspase 3 expression, and cell cycle.

## List of Nonstandard Abbreviations

AJ	Adherens junctions
EC	Endothelial cells
FA	focal adhesion
HUVEC	Human umbilical vein endothelial cells
RFPEC	Rat fat pad endothelial cells
RhoGEF	Rho-specific guanine nucleotide exchange factor
TEM4	Tumor endothelial marker 4
