# Supplementary material for: RhoGEF17—An Essential Regulator of Endothelial Cell Death and Growth
Source: Cells. 2021 Mar 27;10(4):741. doi: 10.3390/cells10040741 (PMC8067313; doi:10.3390/cells10040741)
Supplement: Supplementary file 1 [file cells-10-00741-s001.pdf]

Supplementary Materials to Weber et al. 2021

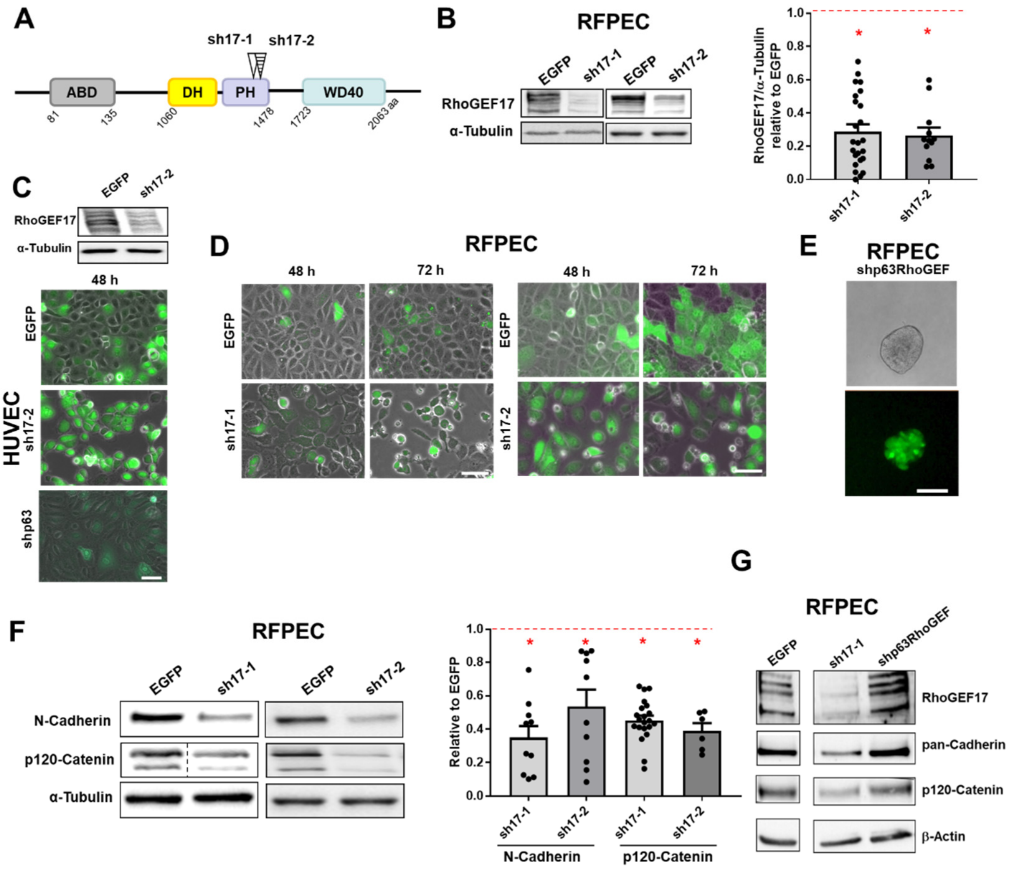

Suppl. Figure 1

Figure S1: RhoGEF17 is essential for cell-cell contacts and AJ protein regulation in EC. A) Shown is the schematic domain structure of RhoGEF17. The positions of the two shRNA target sequences in the PH domain are depicted. ABD = actin binding domain, DH = Dbl homology domain, PH = pleckstrin homology domain, WD40 = domain with seven WD40-related repeats. **B)** RFPEC were transduced for 48 h. Shown are representative immunoblots of RhoGEF17 and α-tubulin and the quantitative analysis. The values were normalized by α-tubulin and are given relative to the respective EGFP controls (not shown). Presented are the means+SEM and the single data points, n=12-24, *p<0.05 vs. the respective EGFP controls assessed by paired t-testing. **C)** Imaging of semi-efficiently transduced HUVEC was performed after 48 h. Depicted are bright field/EGFP overlay images. Scale bar = 100 µm. In addition, an immunoblot of RhoGEF17 and α-tubulin is shown, demonstrating the knockdown efficiency of sh17-2. **D)** Imaging of semi-efficiently transduced RFPEC was performed. Depicted are bright field/EGFP overlay images of different time points.

Scale bar = 100 µm. **E)** RFPEC were transduced for 48 h and then used to generate spheroids. Bright field and fluorescence images are shown. Scale bar = 200 µm. **F)** Immunoblot analysis of N-cadherin, p120-catenin and α-tubulin was performed. Shown are representative immunoblots and the quantitative analysis. The values were normalized by α-tubulin and are given relative to the respective EGFP controls (not shown). Presented are the means+SEM and the single data points, n=6-21, *p<0.05 vs. the respective EGFP controls assessed by t-testing. **G)** Immunoblot analysis of RhoGEF17, pan-cadherin, p120-catenin, and β-actin was performed. Shown are representative immunoblots.

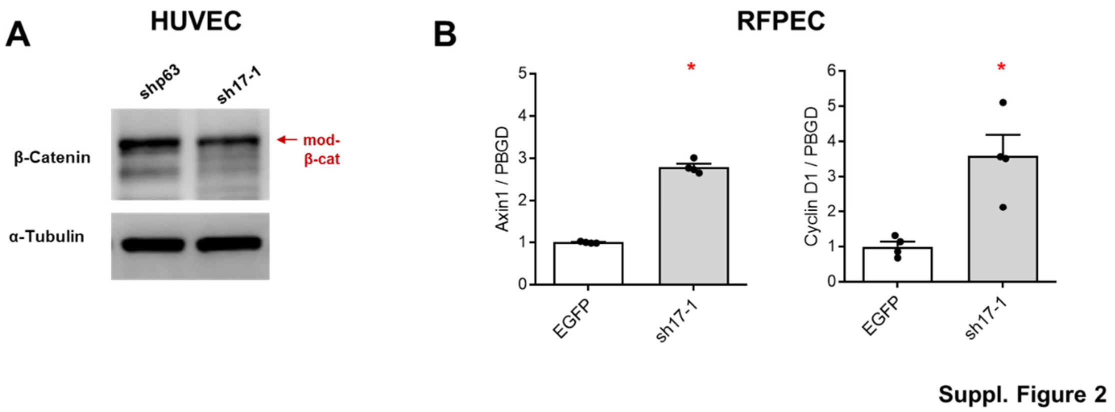

Figure S2: RhoGEF17 regulates β-catenin. A) HUVEC were transduced for 48 h. β-catenin and α-tubulin were detected by immunoblot. **B)** RFPEC were transduced for 48 h and qPCR of axin1, cyclin D1, and the housekeeping gene PBDG was performed. Given are means +SEM of 4 independent experiments, *p<0.05 vs. EGFP assessed by paired t-test.

Primer sequences:

axin 1

forward CCA GTG CCA ATG ACA GTG AG

reverse CCT TCG GTG CTG CTT ACG

cyclin D1

forward CAC CAA TCT CCT CAA CGA C

reverse CAC AGA CCT CCA GCA TCC

PBGD

forward CCT GAAACTCTGCTTCGCTG

reverse CTGGACCATCTTCTTGCTGAA

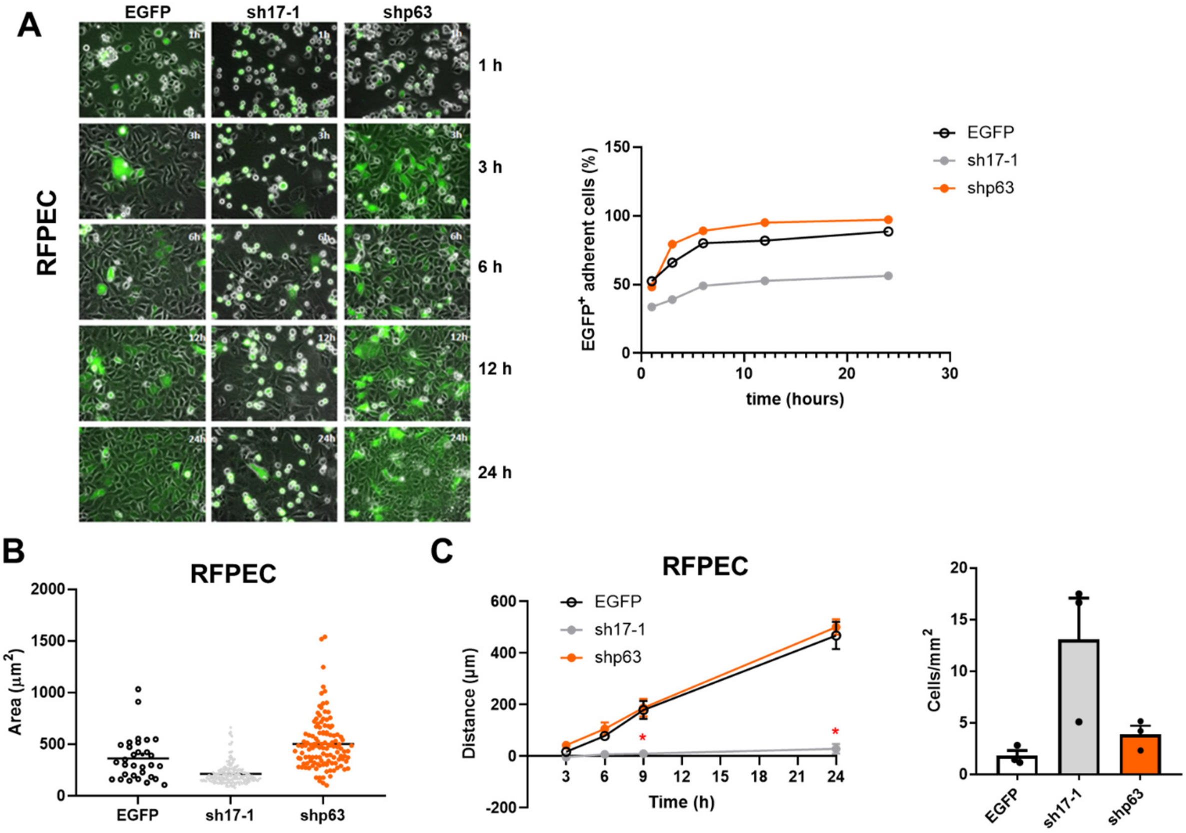

Suppl. Figure 3

Figure S3: The shp63 adenovirus has no effect on RFPEC adhesion, cell size, and sheet migration. RFPEC were transduced for 48 h with the indicated adenoviruses. **A)** The cells were detached and reseeded. Adhesion was monitored by fluorescence microscopy over a time course of 24 h. Depicted are the percentages of transduced ($EGFP^+$), adherent cells given as mean, n=1-2. **B)** The surface area of the cells was determined at the end (24 h) of the adhesion assay. Given are the sizes of the measured cells of 1-2 independent experiments. **C)** Confluent transduced cells were scratched and imaged at the indicated time points. The migration distance of the sheet was measured. Given are the quantified data as mean±SEM, n=3, *p<0.5 vs. EGFP assessed by 2-way ANOVA with Tukey's multiple comparison test. Single, $EGFP^+$ cells in the wound were counted at the end of the assay. The number of cells per mm^2 are given as means+SEM, n=3.

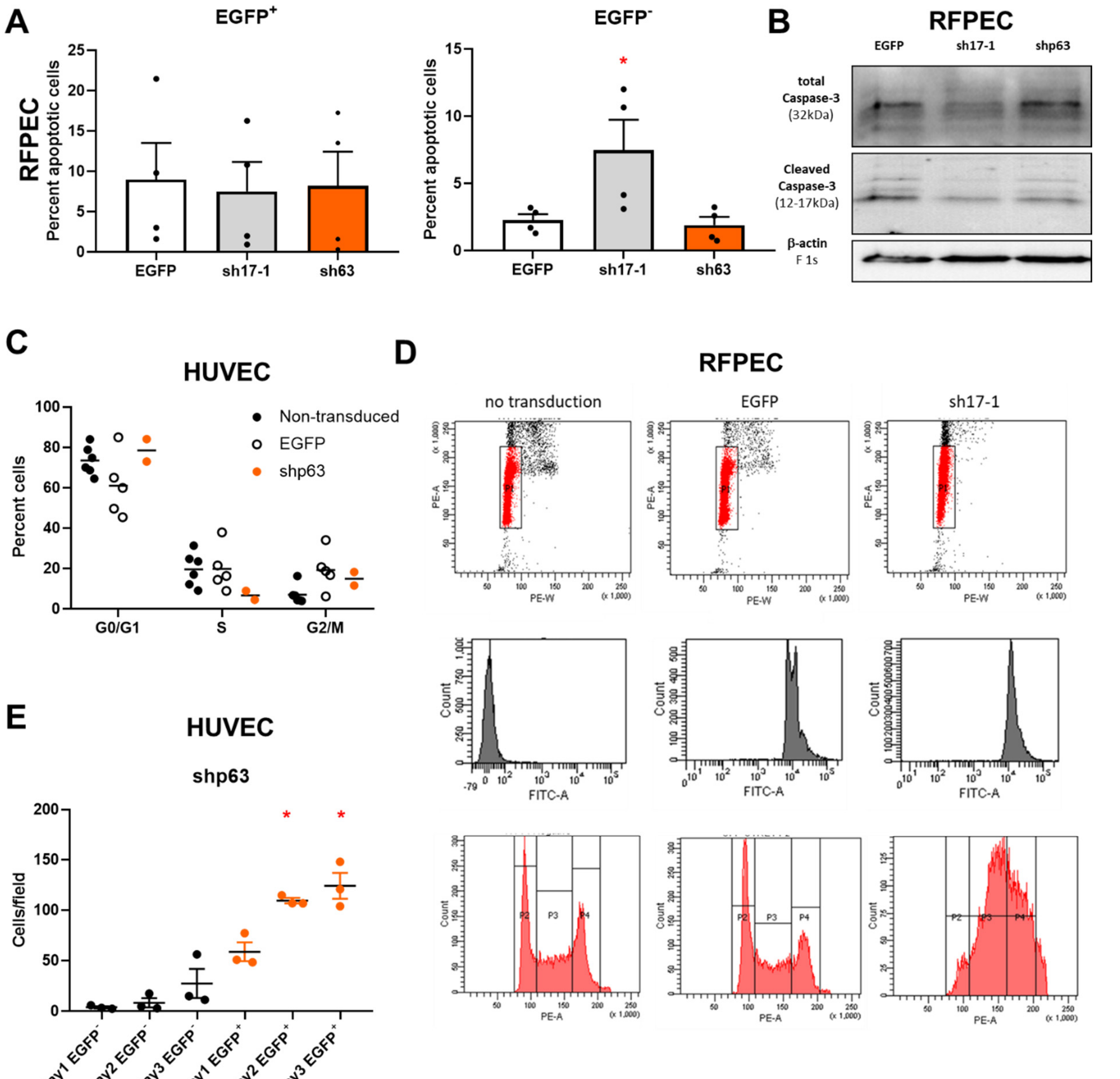

Suppl. Figure 4

Figure S4: The shp63 adenovirus has no effect on EC apoptosis, caspase 3 expression, and cell cycle. RFPEC (A, B, D) or HUVEC (C, E) were transduced with the respective viruses for 48h or the indicated times (E). **A)** Annexin V staining was performed in semi-efficiently transduced cells and Annexin V positive cells were counted and are given in percent of EGFP$^+$ and EGFP$^-$ cells, n=4, *p<0.05 vs. EGFP assessed by 1-way ANOVA with Tukey's post test. **B)** A representative immunoblot of caspase 3 and cleaved caspase 3 together β-actin as loading control is shown. **C-D)** Cell cycle analysis was performed by FACS with PI-stained HUVEC (C) or RFPEC (D). **C)** Analysis of the FACS results for HUVEC, n=2-6. **D)** Cell gating strategy is shown. Upper row: Living single cells, middle row: EGFP-positive cells, bottom row: PI staining vs. cell count. **E)** A proliferation analysis of shp63RhoGEF-transduced HUVEC is presented. EGFP$^-$ and EGFP$^+$ cells were counted from microscope images starting one day after transduction, n=3, *p<0.05 vs. Day1 EGFP$^+$ assessed by 1-way ANOVA with Tukey's post test.
